# Case report of two affected siblings in a family with thiamine metabolism dysfunction syndrome 5: a rare, but treatable neurodegenerative disease

**DOI:** 10.1186/s12883-022-02887-9

**Published:** 2022-09-29

**Authors:** Xiaoyan Li, Zhixin Huang, Yong Chen, Xiaolan Sun, Zhaoshi Yi, Jihua Xie, Xiongying Yu, Hui Chen, Jianmin Zhong

**Affiliations:** Department of Neurology, Jiangxi Provincial Children’s Hospital, Nanchang, 330006 China

**Keywords:** Thiamine metabolism dysfunction syndrome 5, *TPK1*, Thiamine, Case report

## Abstract

**Background:**

Thiamine metabolism dysfunction syndrome 5 (THMD5) is a rare inherited metabolic disorder due to thiamine pyrophosphokinase 1(TPK1) deficiency, caused by mutations in *TPK1*. The core symptoms of the disease is acute or subacute onset encephalopathy, ataxia, muscle hypotonia, and regression of developmental milestones in early infancy, repeatedly triggered by acute infectious illness. However, we report two brothers of THMD5 with compound heterozygous for the mutations c.614-1G > A,c.224 T > A p.(Ile75Asn), but the prognosis is quite different if thiamine suppled. According to our current knowledge, the missense variant c.224 T > A p.(Ile75Asn) was not published previously.

**Case presentation:**

Here, we describe two affected siblings in a Chinese family, after an uneventful pregnancy to non-consanguineous and healthy parents. The older brother presented with normal development during the first 6 months of life, but developed regression of developmental milestones after, accompanied with muscle hypotonia, and chronic encephalopathy, and died at 1 year and 6 months old. The younger brother presented with acute onset encephalopathy, ataxia, muscle hypotonia, repeatedly triggered by acute infectious illness. He was compound heterozygous for the mutations c.614-1G > A,c.224 T > A p.(Ile75Asn) identified by whole exome sequencing. He was diagnosed of THMD5 when he was 11 month. Oral supplementation of thiamine 100 mg/day, the symptoms gradually disappeared. At the age of 2 years and 4 months, he stoped thiamine, his symptoms returned and were once again relieved by oral supplementation of thiamine 100 mg/day.

**Conclusions:**

THMD5 is a rare, but treatable neurodegenerative disease, the clinical phenotype ranges from mild to severe. Massive-dose of thiamine supplementation may ameliorate the course of TPK1 deficiency. When similar clinical cases appear, gene detection is particularly important, which is conducive to early diagnosis. Treatment with thiamine while awaiting the outcome of diagnostic tests may be a good choice.

## Background

Thiamine, also known as vitamin B1, is a water-soluble aromatic substance. It is a crucial cofactor involved in the maintenance of carbohydrate metabolism and participates in multiple cellular metabolic processes within themitochondria, cytosol, and peroxisomes. Many eukaryotes included humans only taken up it from nutrition. Five disorders of thiamine transport or metabolism have been identifed. Thiamine metabolism dysfunction syndrome 1 was caused by *SLC19A2* mutations (coding for thiamine transporter-1 dysfunction), thiamine metabolism dysfunction syndrome 2 was caused by *SLC19A3* mutations (coding for thiamine transporter-1 dysfunction), thiamine metabolism dysfunction syndrome 3 and thiamine metabolism dysfunction syndrome 4 was caused by *SLC25A19* mutations (coding for mitochondrial thiamine pyrophosphate carrier carrier) [1]. Thiamine metabolism dysfunction syndrome 5 (THMD5) is an uncommon subtype of the disorders due to mutations in *TPK1* (coding for thiamine pyrophosphokinase 1, TPK1), which was first described in five patients from three families by Mayr JA et al. in 2011 [2].

Thiamine pyrophosphokinase 1(TPK1) is a cellular enzyme that catalyzes the conversion of thiamine to thiamine pyrophosphateen (TPP), coded by *TPK1* on chromosome 7q35. The length of amino acid of TPR1 is 243. TPP is a cofactor for the pyruvate dehydrogenase complex, branched chain 2-oxo acid dehydrogenase complex, and the oxoglutarate dehydrogenase complex in the mitochondrion. TPP also is a cofactor for the cytosolic transketolase and the peroxisomal 2-hydroxyacyl-CoA lyase 1 in the cytoplasm [1, 3, 4]. Homozygous or compound heterozygous mutations in the *TPK1* gene result in thiamine metabolism dysfunction, and final manifested THMD5 [2].

Here, we report two affected siblings in a family of THMD5, in which the younger brother was treatable with thiamine, but the treatment in older brother did not add thiamine and he died at 1 year and 6 months old. These were rare cases, and we are therefore reporting them to deepen the understanding of this disease and demonstrated the beneficial effect of thiamine.

## Case presentation

**Fig. 1:**
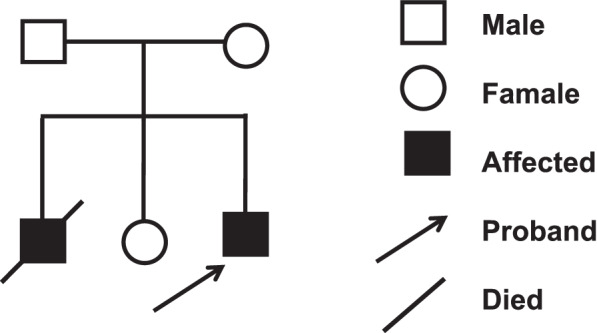
Pedigrees of the affected family

The proband was a boy, born after uneventful pregnancy at term in 2018 (Fig. 1). Parents are nonconsanguineous and healthy. He had a brother and a sister, the older sister is healthy. The older brother presented with normal development during the first 6 months of life, but developed regression of developmental milestones after, accompanied with muscle hypotonia, and chronic encephalopathy. His hypotonia progressed with inconsistent head control, inability to sit without support or walked independently at all until his death at 1 year and 6 months old. The brain magnetic resonance imaging (MRI) was abnormal. No blood sample was available for genetic testing.

The proband showed repeated episodic encephalopathy, triggered by infection diseases. He showed developmental delay and hypotonia. He could turn over at 8 months. He had not raised his head or sit independently, and could only uttered “yiyi” and “yaya” until the first episode occurred at 9 months old.

**Fig. 2:**
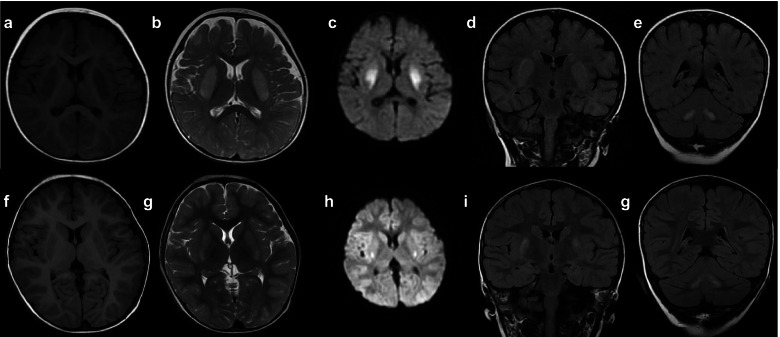
MRI of the brain during the first episode (**a**-**e**) and the second episode (**f**-**g**). af T1WI; bg T2WI; ch DWI; deig T2FLAIR. It showed the abnormal signal in bilateral caudate nucleus, lentiform nucleus, and dentate nucleus

He developed fatigue, weakness, decreased oral intake, lethargy, crying and upset 2 days after an acute upper respiratory tract infection, and did not improve for 4 days, then he was admitted to the Children’s Hospital of Jiangxi Province. Hypermyotonia of his limbs was found when he crying. The babinski reflex was positive on both sides. Results achieved by electroencephalography (EEG), electrocardiography, ultrasound of the abdomen, cerebrospinal fluid (included lactic acid, white blood cell count, level of protein, glucose, chloride, and antibody tests for autoimmune encephalitis), and other biochemical findings were normal (included plasma lactic acid, urinary organic acid metabolism). Brain MRI after admission showed the symmetric high signal on T2-weighted imaging (T2WI) and T2flair in bilateral caudate nucleus, lentiform nucleus, and dentate nucleus, and high signal on difusion weighted imaging (DWI) in the lentiform nucleus and dentate nucleus (Fig. 2a-e).

The patient was diagnosed with suspected encephalitis or congenital genetic metabolic disease. The acyclovir was given to against virus, mannitol was administered to reduce intracranial pressure, immunoglobulin was used to regulation of immune responses, methylprednisolone at high dosage was applied to anti-inflammatory or immunosuppressive action, and vitamin B1(20mg/day), vitamin B6 and vitamin B12 were added. New onset symptoms (included fatigue, weakness, decreased oral intake, lethargy, crying and upset) disappeared on the 10th day after admission, but his developmental milestone did not improved.

**Fig. 3:**
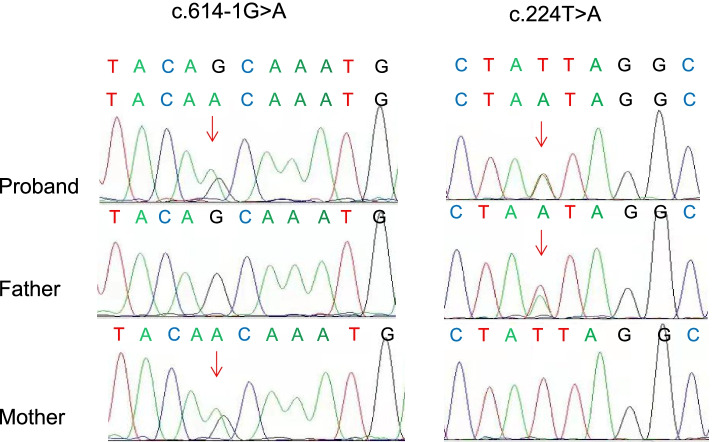
DNA sequence chromatogram of the TPK1 mutations. Arrows indicate the positions of the mutations. A compound heterozygous mutations c.614-1G > A, c.224 T > A p.(Ile75Asn) (NM_022445.3) were found in the proband, the heterozygous missense mutation was found in the parents

**Fig. 4:**
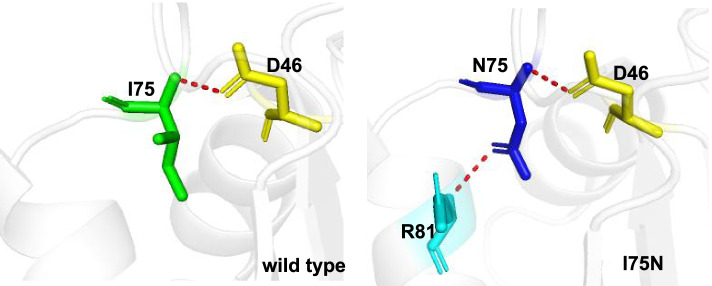
Schematic illustration of the changes in hydrogen bonds. The different amino acids are shown in different colors. The hydrogen bonds are shown as red spheres. The protein modeling showed that mutation Ile75Asn resulting in alteration of hydrogen bonds, that forming a new hydrogen bonds with R81

To further clarify the diagnosis, we conducted mitochondrial DNA sequencing analysis and found no mutation sites with high pathogenicity in mitochondrial DNA. Whole-exome next-generation sequencing (NGS) of the nuclear genome revealed a compound heterozygous for the mutations c.614-1G > A, c.224 T > A p.(Ile75Asn) when he was 11 months old. Sanger sequencing of the parents was performed and confirmed compound heterozygosity of the two variants in the patient (Fig. 3). It confirmed that the older sisiter was heterozygote. The mutations were not present in the controls of gnomAD-East Asian and gnomAD-all population. Mutations c.614-1G > A occurred at a splicing site and led to the abnormal formation of TPP, and it was published previously in a Chinese female patient [5], confirming its pathogenicity. All the reported mutations were retrieved from PubMed (https://pubmed.ncbi.nlm.nih.gov/) and Human Gene Mutation Database (http://www.hgmd.cf.ac.uk/ac/index.php) up to March 2021. The missense mutation c.224 T > A p.(Ile75Asn) was not published before. It was predicted as being damaging by in silico tools (http://varcards.biols.ac.cn/). The molecular effect of the missense mutations was analyzed by protein modeling using PHYRE2 (http://www.sbg.bio.ic.ac.uk/~phyre2/html/page.cgi?id=index) and PyMOL with the templates available (Fig. 4). The protein modeling showed that mutation Ile75Asn resulted in alteration of hydrogen bonds and potentially affected the protein steric configuration. Originally, residue Ile75 formed one hydrogen bonds with residue D46, When isoleucine was replaced by asparagine at residue Ile75, the hydrogen bonds with residue D46 was remained, but form a new hydrogen bonds with R81.

Vitamin B1(100mg/day in 2 daily doses) was subsequently administered every day, and other medications have been stoped. After a few months, hypotonia disappeared, he was able to raised his head, sit independently and communicated with words. He could walk independently at 1 year and 9 months. At this point, 17 months after diagnosis, no more encephalopathic episodes have occurred under ongoing supplementation with vitamin B1.

The vitamin B1 was stoped at 2 years 4 months by his parents, when the developmental milestone was basically consistent with peers. But 10 days later, he developed a second episode after an acute viral infection. He became limply, unable to walk, decreased oral intake. Disturbance of consciousness (he did not recognize anyone and he couldn’t speak), burst out crying, hypermyotonia of his limbs, fever and dyspnea were observed 2 days later, he was admitted to the Children’s Hospital of Jiangxi Province again. Plasma lactic acid is 4.1 mmol/L. Repeated brain MRI showed the symmetric high signal on T2WI and T2flair in bilateral lentiform nucleus and dentate nucleus, and high signal on DWI in the lentiform nucleus (Fig. 2f-g). Lesion sites were significantly improved during MRI examination. Vitamin B1(100mg/day in 2 daily doses) was administered again. These symptoms returned to normal after mannitol, oxygen, sodium bicarbonate and vitamin B1 supplementation about 7 days later. No recurrence was found during follow-up.

## Discussion and conclusion

The older brother presented with developed regression, muscle hypotonia, chronic encephalopathy, and early death. The younger brother presented with acute onset encephalopathy, ataxia, muscle hypotonia, repeatedly triggered by acute infectious illness, massive-dose thiamine was effective. The two patients from one family, presented with THMD5 as published before, but the prognosis is totally different.

**Fig. 5:**
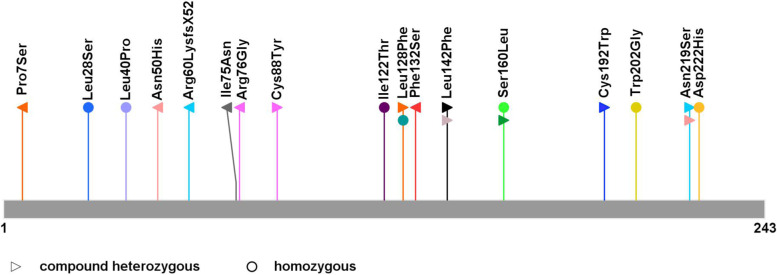
Schematic diagram of 17 missense TPK1 mutations and their locations on TPK1 protein

Here, we report a pair of compound heterozygous mutations in the *TPK1.*Although the DNA of older brother was not available for testing, his clinical characteristics and family-based linkage analysis suggested he had THMD5 too. We reviewed the previous studies through Human Gene Mutation Database and PubMed, and analyzed the genotype and phenotype of *TPK1* mutations and THMD5. According to our current knowledge, the missense variant c.224 T > A p.(Ile75Asn) was not published previously. Previously and our cases, 19 genotypes were reported in 28 patients, including 9 homozygous mutations and 10 compound heterozygous mutations, in which 17 missense variants (Fig. 5), 2 frameshift variants (c.[179_182delGAGA], c.311delG), 1 microdeletion (deletion of exons 3 and 4), and 3 splice site variants (c.[258 + 1G > A], c.501 + 4A > T, and c.614-1G > A) [2, 6–18]. Most of the variations come from heterozygous and phenotypically normal parents，especially in consanguineous marriage. The siblings of patients are more likely to be attacked by the disease, such as the brothers in our study.

THMD5 is a rare neurodegenerative disorder. We reviewed 15 related publications on THMD5 and analyzed the prevalence of major medical issues from 28 patients including two patients in this family (14 males, 13 female, one unknown) [2, 6–18]. Most patients manifested as Leigh-like syndrome, recurrent episodic acute or subacute encephalopathy. Core symptoms of THMD5 include developmental delay (5/23), developed regression (9/23), dystonia (12/23), hypotonia (7/22), seizure/epilepsy (6/22), gait ataxia (10/22), consciousness disorders (9/14), and triggered by acute infectious illness (20/23). Only one patient was reported to have dyspnea to date [2, 4, 8, 9]. All described patients had onset of symptoms from 1 month to 4 years and 8 months. The patients died from 6 months to 8 years and 6 months if thiamine was not added [2, 4, 8, 9]. The brain MRI were abnormal in most cases (17/21). The principal manifestations of the MRI include abnormal signal in the basal ganglia, cerebellum, brainstem and global atrophy [14]. It is symmetric or asymmetric. The affected siblings in our study presented with the above major symptoms and MRI manifestation. But the pathogenic progression of the two brothers are completely different. In addition, dyspnea was discovered in our case. Mahajan A et al. and Invernizzi F had reported cases who were initially misdiagnosed as acute disseminated encephalomyelitis and received corticosteroid and intravenous gamma globulins, so as the proband in this study [6, 16]. All but one patients have been confirmed the diagnosis by next-generation sequencing. Invernizzi F et al. reported a patient with a strong clinical suspicion, he found a heterozygous mutation by genomic DNA mutations, then found the allele with exon 3 and 4 deletion by mRNA sequencing [16].

Until now, thiamine supplementation (100-750 mg/day) were reported in 18 patients [2, 5–10, 16–18], 11 patients (11/18) has led to clinical improvement and even normal neurodevelopment, some accompanied by prevent further metabolic decompensations and ameliorate brain MRI lesions. In these cases, the older brother was untreated with thiamine, and he was died at 1 year and 6 months. The younger brother was treated with thiamine (100 mg/day), he showed with clinical improvement and even normal neurodevelopment after a few months. But when the thiamine was stoped 10 days, about a half-life of thiamine (9–18 days), he had a similar episodes. Then symptoms were reversible after thiamine taked again. Brain MRI lesions had significantly improved after massive-dose thiamine treatment. This study proves that thiamine is crucial, maybe dose-dependent, and is effective in the THMD5. Seven patients (7/18) showed no clinical improvement after thiamine suppled. We speculate that the probable reasons including the later time of started thiamine suppled, the more severe of the clinical manifestations before initiation of treatment，and the lower doses of thiamine applied, but no patient was reported died after thiamine suppled.

In conclusion, this study identified a compound heterozygous for the *TPK1* mutations in a Chinese family including two patients, but the prognosis is quite different if thiamine suppled. Early diagnosis and effective treatment regimens are the keys in the THMD5.When similar clinical cases appear, gene detection is particularly important, and treatment with thiamine while awaiting the outcome of diagnostic tests may be a good choice.

## Data Availability

The datasets used and/or analysed during the current study are available from the corresponding author on reasonable request.

## Abbreviations

THMD5: Thiamine metabolism dysfunction syndrome 5
TPK1: Thiamine pyrophosphokinase 1
TPP: Thiamine pyrophosphateen
MRI: Magnetic resonance imaging
EEG: Electroencephalography
T2WI: T2-weighted imaging
DWI: Difusion weighted imaging
NGS: Next-generation sequencing

## References

[1] Marcé-Grau A, Martí-Sánchez L, Baide-Mairena H (2019). Genetic defects of thiamine transport and metabolism: a review of clinical phenotypes, genetics, and functional studies. J Inherit Metab Dis.

[2] Mayr JA, Freisinger P, Schlachter K (2011). Thiamine pyrophosphokinase deficiency in encephalopathic children with defects in the pyruvate oxidation pathway. Am J Hum Genet.

[3] Brown G (2014). Defects of thiamine transport and metabolism. J Inherit Metab Dis.

[4] Ortigoza-Escobar JD, Alfadhel M, Molero-Luis M (2017). Thiamine deficiency in childhood with attention to genetic causes: survival and outcome predictors. Ann Neurol.

[5] Li D, Song J, Li X (2020). Eleven novel mutations and clinical characteristics in seven Chinese patients with thiamine metabolism dysfunction syndrome. Eur J Med Genet.

[6] Mahajan A, Sidiropoulos C (2017). TPK1 mutation induced childhood onset idiopathic generalized dystonia: report of a rare mutation and effect of deep brain stimulation. J Neurol Sci.

[7] Banka S, de Goede C, Yue WW (2014). Expanding the clinical and molecular spectrum of thiamine pyrophosphokinase deficiency: a treatable neurological disorder caused by TPK1 mutations. Mol Genet Metab.

[8] Fraser JL, Vanderver A, Yang S (2014). Thiamine pyrophosphokinase deficiency causes a Leigh disease like phenotype in a sibling pair: identification through whole exome sequencing and management strategies. Mol Genet Metab Rep.

[9] Huang W, Qin J, Liu D (2018). Reduced thiamine binding is a novel mechanism for TPK deficiency disorder. Mol Genet Genomics.

[10] Zhu L, Ruijuan W, Ye Z (2019). Identification of two novel TPK1 gene mutations in a Chinese patient with thiamine pyrophosphokinase deficiency undergoing whole exome sequencing. J Pediatr Endocrinol Metab.

[11] Hu C, Li X, Zhao L (2020). Clinical and molecular characterization of pediatric mitochondrial disorders in south of China. Eur J Med Genet.

[12] Rüsch CT, Wortmann SB, Kovacs-Nagy R (2021). Thiamine Pyrophosphokinase deficiency due to mutations in the TPK1 gene: a rare, , Treatable Neurodegenerative Disorder. Neuropediatrics.

[13] Eckenweiler M, Mayr JA, Grünert S (2021). Thiamine treatment and favorable outcome in an infant with Biallelic TPK1 variants. Neuropediatrics..

[14] Zhu B, Wu J, Chen G (2020). Whole exome sequencing identifies a novel mutation of TPK1 in a Chinese family with recurrent Ataxia. J Mol Neurosci.

[15] Ortigoza-Escobar JD, Alfadhel M, Molero-Luis M (2017). Thiamine deficiency study group. Thiamine deficiency in childhood with attention to genetic causes: survival and outcome predictors. Ann Neurol.

[16] Invernizzi F, Panteghini C, Chiapparini L (2017). Thiamine-responsive disease due to mutation of tpk1: importance of avoiding misdiagnosis. Neurology..

[17] Nyhan WL, McGowan K, Barshop BA (2019). Thiamine phosphokinase deficiency and mutation in TPK1 presenting as biotin responsive basal ganglia disease. Clin Chim Acta.

[18] Bugiardini E, Pope S, Feichtinger RG (2019). Utility of whole blood thiamine pyrophosphate evaluation in TPK1-related diseases. J Clin Med.

